# Supplementary material for: Fat and Carbohydrate Interact to Potentiate Food Reward in Healthy Weight but Not in Overweight or Obesity
Source: Nutrients. 2021 Apr 6;13(4):1203. doi: 10.3390/nu13041203 (PMC8067354; doi:10.3390/nu13041203)
Supplement: Supplementary file 1 [file nutrients-13-01203-s001.pdf]

nutrients

MDPI

Article

Fat and carbohydrate interact to potentiate food reward in healthy weight but not in overweight or obesity

Supplementary Materials

Table S1. Descriptive statistics for subjective ratings and willingness to pay (WTP) for foods across the three macronutrient categories from participants with healthy weight (HW; n = 30).

**Subjective Variable** (Units or scale range)	**Carbohydrate** M ± SD, *Range*	**Fat** M ± SD, *Range*	**Combo** M ± SD, *Range*
Liking (-100 to +100%)	18.6 ± 31.4, *-100 to +100*	17.3 ± 32.0, *-100 to +83.0*	20.3 ± 36.1, *-100 to +89.3*
Familiarity (0-100%)	86.0 ± 22.3, *0-100*	79.7 ± 26.3, *0-100*	83.5 ± 22.2, *0-100*
Frequency (days/month)	2.40 ± 3.42, *0-15.04*	2.83 ± 3.84, *0-20*	2.31 ± 3.33, *0-15.29*
Expected satiety (0-100%)	42.3 ± 26.0, *0-100*	59.1 ± 19.4, *0-100*	47.1 ± 23.0, *0-100*
Healthiness (0-100%)	36.7 ± 24.3, *0-100*	46.5 ± 20.0, *0-100*	35.9 ± 22.3, *0-94.1*
Estimated energy content (kcal)	115 ± 50, *17-240*	122 ± 45, 21-*240*	110 ± 46, 0-*240*
Estimated energy density (0-100%)	49.0 ± 24.5, *0-100*	66.8 ± 17.2, *0-100*	55.7 ± 22.4, *0-100*
Estimated price (USD)	1.34 ± 0.96, *0.07-5*	1.57 ± 1.11, *0-5*	1.29 ± 0.99, *0.06-5*
Willingness to pay (USD)	0.94 ± 0.96, *0-4.92*	0.98 ± 1.09, *0-5*	1.04 ± 0.95, *0-4.55*

Table S2. Descriptive statistics for subjective ratings and WTP for foods across the three macronutrient categories from participants with overweight/obesity (OW/OB; n = 30).

**Subjective Variable** (Units or scale range)	**Carbohydrate** M ± SD, *Range*	**Fat** M ± SD, *Range*	**Combo** M ± SD, *Range*
Liking (-100 to +100%)	17.1 ± 32.4, *-90.4 to +100*	17.6 ± 31.0, *-95.4 to +100*	21.6 ± 30.4, *-86.9 to +100*
Familiarity (0-100%)	80.3 ± 24.0, *0-100*	75.7 ± 28.0, *0-100*	78.6 ± 24.6, *0-100*
Frequency (days/month)	2.28 ± 3.57, *0-20*	3.00 ± 4.12, *0-20*	2.05 ± 2.82, *0-10.81*
Expected satiety (0-100%)	43.5 ± 24.3, *0-100*	55.8 ± 18.4, *0-100*	43.5 ± 21.0, *0-94.9*
Healthiness (0-100%)	37.4 ± 23.5, *0-94.6*	48.3 ± 18.0, *0-100*	35.9 ± 20.2, *0-81.4*
Estimated energy content (kcal)	128 ± 52, *18-240*	121 ± 48, *0-240*	122 ± 53, *0-240*
Estimated energy density (0-100%)	50.3 ± 22.0, *0-100*	62.1 ± 14.6, *18.1-100*	53.2 ± 30.9, *0-100*
Estimated price (USD)	1.51 ± 0.99, *0.06-5*	1.82 ± 1.10, *0.04-5*	1.50 ± 0.97, *0.06-5*
Willingness to pay (USD)	1.07 ± 0.98, *0-4.38*	1.15 ± 1.07, *0-5*	1.09 ± 0.98, *0-4.89*

Table S3. Descriptive statistics and unpaired, two-sample t-tests on the Dietary Fat and Free Sugar Short Questionnaire (DFS) subscores and internal state ratings across BMI groups.

**DFS Score or Internal State** (Scale range)	**HW** M ± SD, *Range*	**OW/OB** M ± SD, *Range*	**BMI Group Difference**
DFS fat (0-55)	28.1 ± 5.7, *19-43*	28.7 ± 6.0, *19-46*	$t_{(1,58)} = 0.379$, $p = 0.706$
DFS sugar (0-45)	18.7 ± 4.6, *11-29*	18.2 ± 5.3, *9-32*	$t_{(1,58)} = -0.346$, $p = 0.731$
DFS fat-sugar (0-30)	13.6 ± 3.3, *7-20*	12.7 ± 3.2, *9-22*	$t_{(1,58)} = -1.143$, $p = 0.258$
Hunger (0-100%)	71.5 ± 18.4, *0.1-97.4*	67.1 ± 23.2, *0-99.4*	$t_{(1,58)} = -0.819$, $p = 0.416$
Fullness (0-100%)	23.9 ± 20.9, *0-84.7*	23.2 ± 19.5, *0-66.0*	$t_{(1,58)} = -0.122$, $p = 0.903$
Thirst (0-100%)	69.4 ± 20.0, *25.4-100*	73.2 ± 19.9, 20.0-*100*	$t_{(1,58)} = 0.751$, $p = 0.456$
Desire to eat (0-100%)	72.1 ± 17.3, *26.7-98.9*	66.4 ± 18.4, *25.9-100*	$t_{(1,58)} = -1.241$, $p = 0.220$

Potential to eat (0-100%)	71.4 ± 16.5, *26.5-100*	66.4 ± 14.3, 26.5-*98.0*	$t_{(1,58)} = -1.257$, $p = 0.214$

Table S4. BMI group interactions in the regressions between WTP, actual energy density (AED), and estimated energy density (EED), tested on averages per food across participants with HW versus OW/OB.

Variables	Foods Included	BMI Group Interaction
**Outcome: WTP**	All stimuli	β = 0.104, p = 0.627
**Predictors: AED × BMI Group**	Carbohydrate items	β = -0.073, p = 0.858
	Fat items	β = 0.159, p = 0.690
	Combo items	β = 0.351, p = 0.262
**Outcome: WTP**	All stimuli	β = 0.017, p = 0.511
**Predictors: EED × BMI Group**	Carbohydrate items	β = 0.018, p = 0.736
	Fat items	β = 0.011, p = 0.885
	Combo items	β = 0.012, p = 0.838
**Outcome: AED**	All stimuli	β = -0.018, p = 0.480
**Predictors: EED × BMI Group**	Carbohydrate items	β = -0.006, p = 0.897
	Fat items	β = -0.028, p = 0.748
	Combo items	β = -0.061, p = 0.206

Table S5. Correlations between actual energy density (AED) and characteristics and subjective ratings of foods in the combo category, tested on averages per food item across all participants.

Variable	Correlation with AED
Liking	$r^2 = 0.392$, $p = 0.029$
Familiarity	$r^2 = 0.002$, $p = 0.884$
Frequency	$r^2 = 0.327$, $p = 0.052$
Healthiness	$r^2 = 0.637$, $p = 0.002$ *
Expected satiety	$r^2 = 0.671$, $p = 0.001$ *
Estimated energy content	$r^2 = 0.229$, $p = 0.115$
Estimated energy density	$r^2 = 0.587$, $p = 0.004$
Estimated price	$r^2 = 0.835$, $p < 0.001$ *
Actual price	$r^2 = 0.859$, $p < 0.001$ *
Volume	$r^2 = 0.703$, $p < 0.001$ *
Visual area	$r^2 = 0.052$, $p = 0.478$
Fat content	$r^2 = 0.062$, $p = 0.436$
Carbohydrate content	$r^2 = 0.067$, $p = 0.417$
Protein content	$r^2 = 0.343$, $p = 0.046$
Sodium content	$r^2 = 0.550$, $p = 0.006$

* p < 0.0033 after Bonferroni correction for the 15 correlations performed

Table S6. Testing if the relationships of food volume and expected satiety with actual energy density differ across pairwise comparisons of foods in the three macronutrient categories.

Variables	Pairwise Comparison	Macronutrient Category Difference
**Outcome: AED**	Carbohydrate versus Fat	$t_{(1,20)} = 2.785$, $p = 0.011$ *
**Predictors: Volume × Macronutrient**	Carbohydrate versus Combo	$t_{(1,20)} = 1.166$, $p = 0.257$
	Fat versus Combo	$t_{(1,20)} = 1.229$, $p = 0.233$
**Outcome: AED**	Carbohydrate versus Fat	$t_{(1,20)} = 2.478$, $p = 0.022$
**Predictors: Expected Satiety × Macronutrient**	Carbohydrate versus Combo	$t_{(1,20)} = 2.45$, $p = 0.024$
	Fat versus Combo	$t_{(1,20)} = 2.211$, $p = 0.039$

* p < 0.017 after Bonferroni correction for the 3 pairwise comparisons per combination of outcome and predictor variables.

Table S7. Participant characteristics of the independent cohort for testing the modified picture set with varying food portions (N = 22).

**Characteristic** (Units)	**Mean ± SD, *Range***
Sex	6 Male, 16 Female
Age (yr)	22.3 ± 6.6, *18-45*
Education (yr)	14.5 ± 2.5, *12-20*
Race	12 White, 1 Black/African American, 8 Asian, 1 More than one race
Ethnicity	2 Hispanic or Latino, 20 Not Hispanic or Latino
Household income [1]	5.2 ± 2.2, *1-8*
Height (m)	1.68 ± 0.07, *1.58-1.79*
Weight (kg)	62.35 ± 7.61, *48.10-77.25*
Body mass index (kg/m²)	22.04 ± 1.72, *18.42-24.94*
Waist-hip ratio	0.84 ± 0.07, *0.72-1.05*
Body fat (%)	23.5 ± 7.1, *9.5-33.8*

[1] Household income was dummy coded from 1-8 according to 2018 US Census Bureau income percentiles.

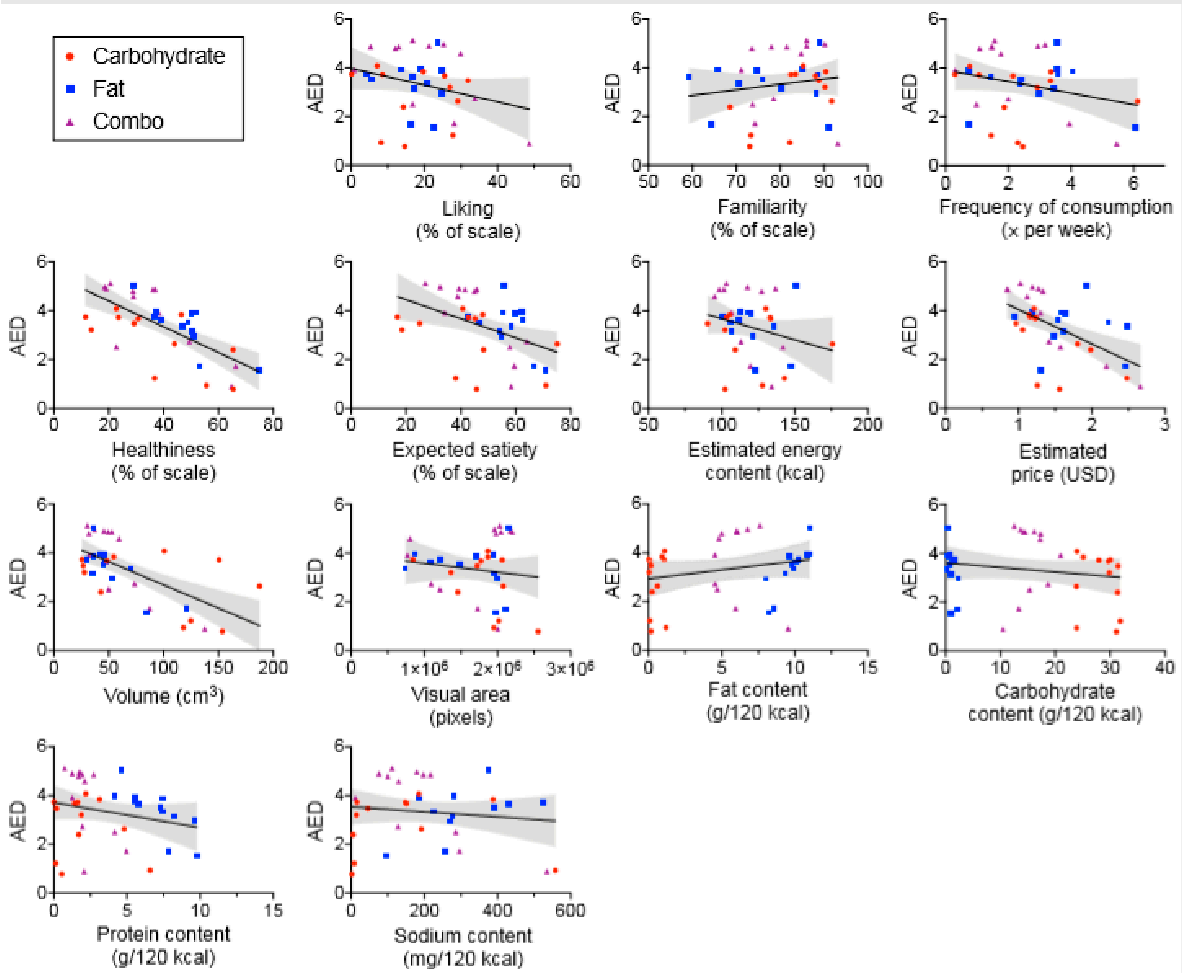

Figure S1. Fitted scatter plots comparing each food characteristic and subjective rating with actual energy density (AED, in g/120 kcal). Each data point represents a single food item from one of the three macronutrient categories (carbohydrate, fat, combo), with ratings averaged across all N = 60 participants (n = 30 HW and n = 30 OW/OB). Shading indicates 95% CI for the line of best fit.

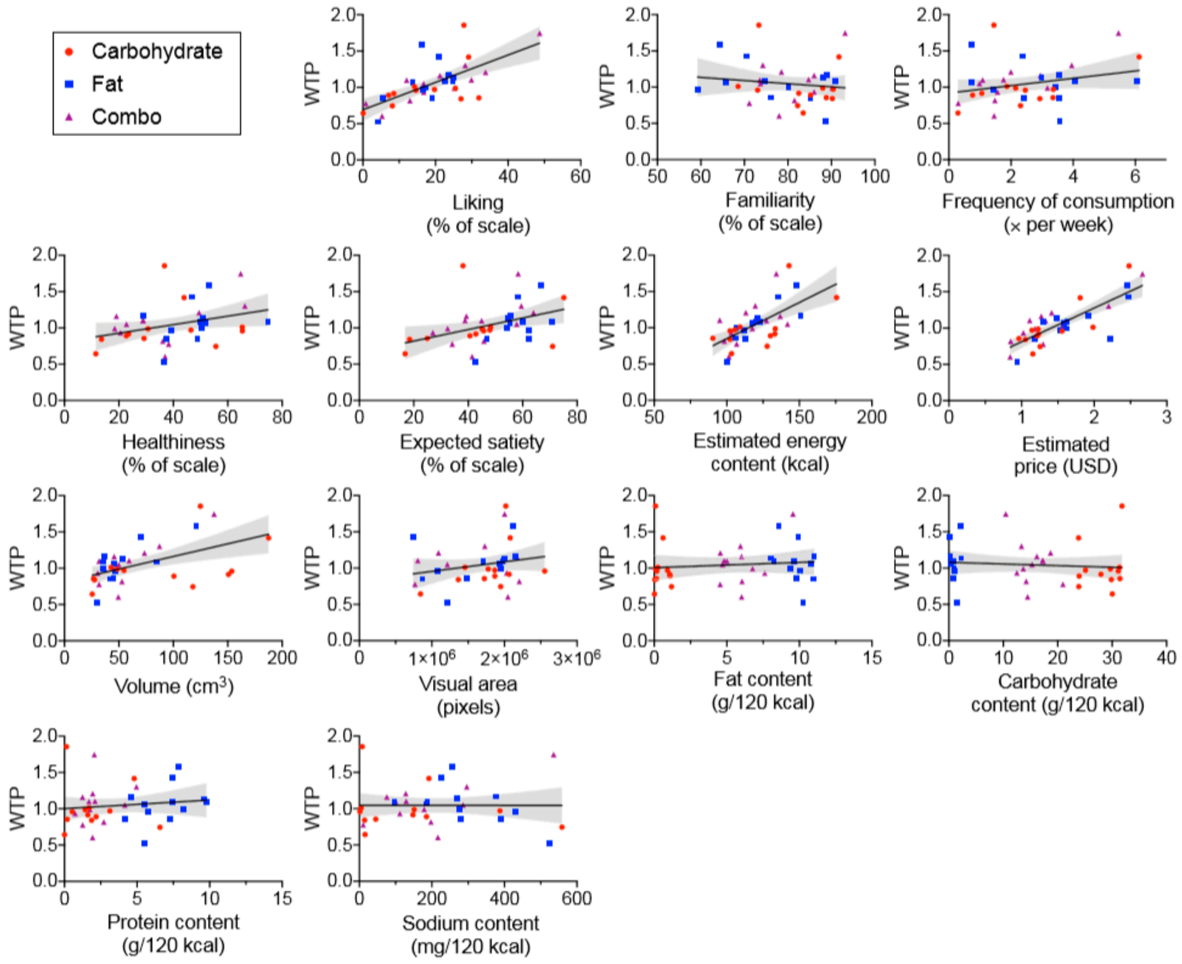

Figure S2. Fitted scatter plots comparing each food characteristic and subjective rating with willingness to pay (WTP, in USD). Each data point represents a single food item from one of the three macronutrient categories (carbohydrate, fat, combo), with ratings averaged across all N = 60 participants (n = 30 HW and n = 30 OW/OB). Shading indicates 95% CI for the line of best fit.

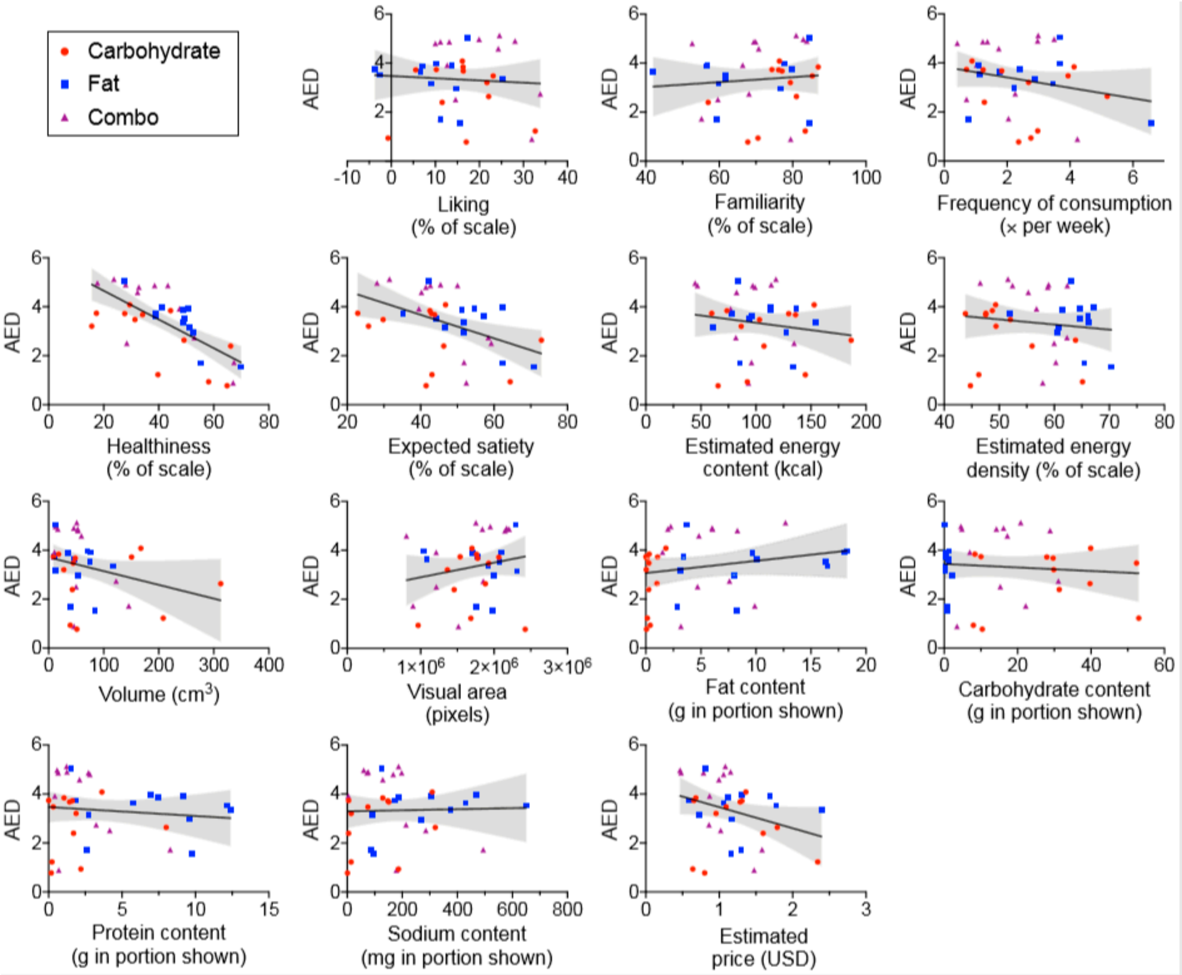

Figure S3. Fitted scatter plots comparing each characteristic and subjective rating with actual energy density (AED, in g/120 kcal) for foods pictured in varying portions (40, 120, 200 kcal). Each data point represents a single food item from one of the three macronutrient categories (carbohydrate, fat, combo), with ratings averaged across all N = 22 participants of the independent sample. Shading indicates 95% CI for the line of best fit.

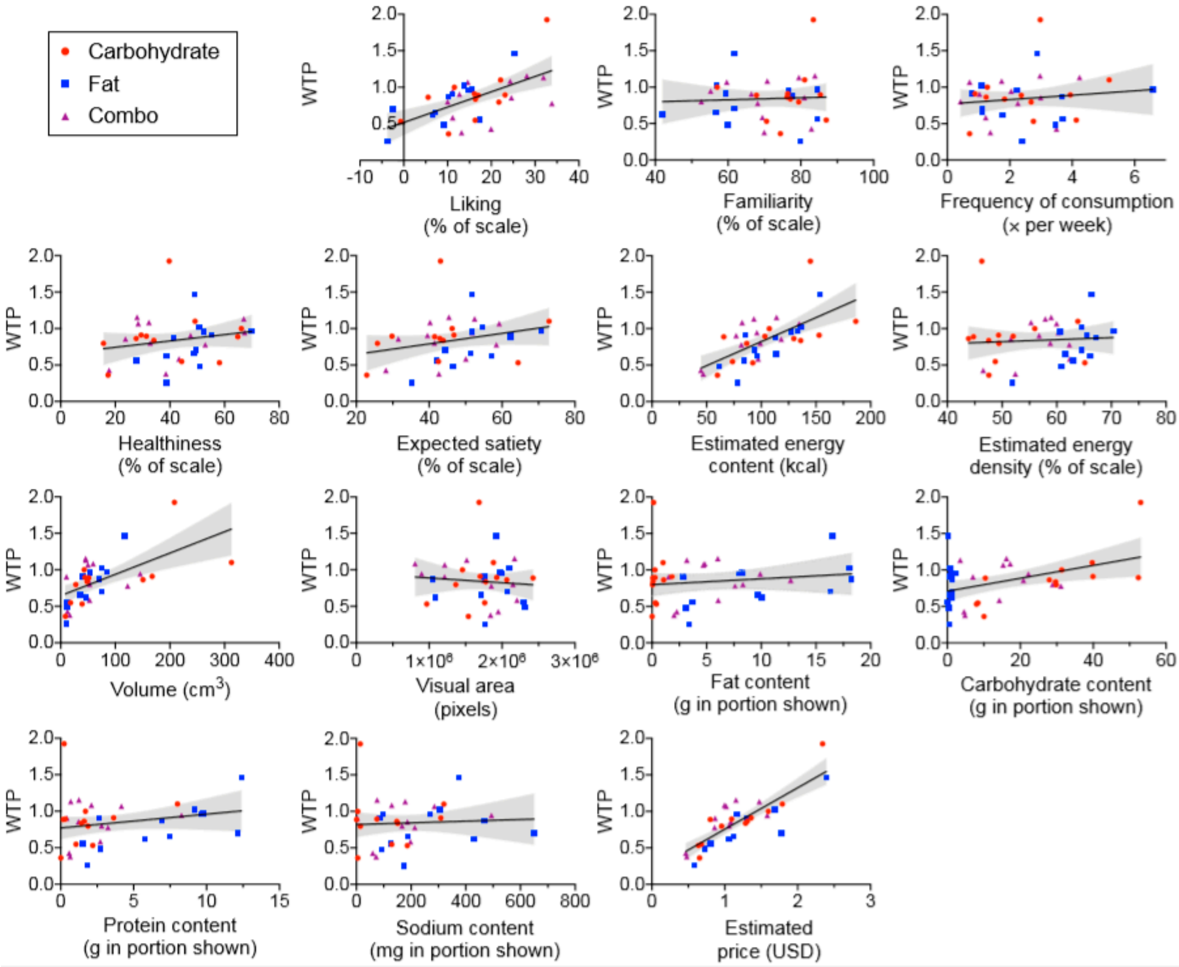

Figure S4. Fitted scatter plots comparing each characteristic and subjective rating with willingness to pay (WTP, in USD) for foods pictured in varying portions (40, 120, 200 kcal). Each data point represents a single food item from one of the three macronutrient categories (carbohydrate, fat, combo), with ratings averaged across all N = 22 participants of the independent sample. Shading indicates 95% CI for the line of best fit.
